# Extracorporeal shock waves down-regulate the expression of interleukin-10 and tumor necrosis factor-alpha in osteoarthritic chondrocytes

**DOI:** 10.1186/1471-2474-9-16

**Published:** 2008-01-31

**Authors:** Biagio Moretti, Florenzo Iannone, Angela Notarnicola, Giovanni Lapadula, Lorenzo Moretti, Vittorio Patella, Raffaele Garofalo

**Affiliations:** 1 Department of Clinical Methodology and Surgical Technique, Orthopaedics Section, University of Bari, Bari, Italy; 2 Department of Internal Medicine and Public Medicine, Rheumatology Unit, University of Bari, Bari, Italy

## Abstract

**Background:**

The purpose of this study was to investigate the effects of extra corporeal shock waves (ESW) therapy on the metabolism of healthy and osteoarthritic human chondrocytes, and particularly on the expression of IL-10, TNF-alpha and beta1 integrin.

**Methods:**

Human adult articular cartilage was obtained from 9 patients (6 male and 3 females), with primary knee osteoarthritis (OA), undergoing total joint replacement and from 3 young healthy donors (HD) (2 males, 1 female) with joint traumatic fracture. After isolation, chondrocytes underwent ESW treatment (electromagnetic generator system, MINILITH SL1, STORZ MEDICAL) at different parameters of impulses, energy levels and energy flux density. After that, chondrocytes were cultured in 24-well plate in DMEM supplemented with 10% FCS for 48 hours and then beta_1 _integrin surface expression and intracellular IL-10 and TNF-alpha levels were evaluated by flow-cytometry.

**Results:**

At baseline, osteoarthritic chondrocytes expressed significantly lower levels of beta1 integrin and higher levels and IL-10 and TNF-alpha levels. Following ESW application, while beta1 integrin expression remain unchanged, a significant decrease of IL-10 and TNF-alpha intracellular levels was observed both in osteoarthritic and healthy chondrocytes. IL-10 levels decreased at any impulses and energy levels, while a significant reduction of TNF-alpha was mainly found at middle energies.

**Conclusion:**

Our study confirmed that osteoarthritic chondrocytes express low beta_1 _integrin and high TNF-alpha and IL-10 levels. Nonetheless, ESW treatment application down-regulate the intracellular levels of TNF-alpha and IL-10 by chondrocytes, suggesting that ESW might restore TNF-alpha and IL-10 production by osteoarthritic chondrocytes at normal levels. However, further in vivo and in vitro studies are necessary to establish if ESW can represent a viable option in the treatment of OA.

## Background

Extra-corporeal shock waves (ESW) are expanding their applications from urinary calculi treatment to orthopaedic settings. Recent studies have provided some evidence that ESW may be useful in treating osteoarthritis (OA) in animals, such as dogs [1], and horses [2]. In humans, ESW therapy is widely used for the treatment of several medical disorders such as plantar fascitis, calcifying tendonitis, femoral head necrosis and pseudoarthrosis, and more recently has been proposed as therapy for human OA. However, whether and how ESW hamper the biologic processes taking place in articular cartilage that cause OA is unknown.

In OA, cartilage damage is the outcome of an abnormal extra-cellular matrix (ECM) remodelling leading to an overwhelm of tissue breakdown mediated by metalloproteinases (MMPs). The pathogenesis of OA is rather intricate and not yet completely understood, however some events are currently assumed to be critical points in the induction of cartilage injury [3]. Under physiological settings, chondrocytes sense the changes of surrounding environment and this signalling between chondrocytes and ECM is crucial in maintaining cartilage homeostasis. These chondrocyte/ECM interactions are regulated by a large family of transmembrane glycoproteins whereby beta_1 _integrins are the most widely expressed and interact with several matrix proteins such as collagen, fibronectin, vitronectin and laminin [4]. Furthermore, beta_1 _integrin cooperate along with growth factor receptors (GFr), such as TGFbeta, IL-4, and IGF, to activate intracellular anabolic processes [4]. In OA, beta_1 _integrin expression is significantly reduced in damaged cartilage [5] and this would alter chondrocyte/ECM interactions and disrupt beta_1 _integrin/GFr synergy leading to activation of catabolic pathways, increase of MMPs expression and induction of chondrocyte apoptosis.

The over-expression of MMPs is induced by several cytokines, such as IL-1, TNF-alpha, IL-17, IL-10, and many others, which have been detected increased in OA cartilage. TNF-alpha is produced by inflamed synovial membrane, chondrocytes and osteoblasts, and act in an autocrine-paracrine manner. It increases the synthesis of MMPs and plasminogen activator, essential to convert the pro-MMPs in MMPs, and regulate the organization of ECM by enhancing the synthesis of minor collagens, normally not present in cartilage, such as collagen types I and III, and decreasing the production of proteoglycans and collagen types II and IX, which represent the optimal scaffolding of cartilage [3]. The biological activities of TNF-alpha are regulated by two specific cell-surface receptors TNF-R_55 _and TNF-R_75_; the former is mainly involved in signal transduction in articular tissue cells and its expression is up-regulated on osteoarthritic chondrocytes and synoviocytes, that in turn show an increased sensitivity to TNF-alpha [6,7].

IL-10 activates a broad range of functional responses in different cell types inducing either inhibitory or stimulatory effects, such as down-regulating IL-1 and TNF-alpha synthesis by monocytes [8] or promoting the growth and differentiation of B cells [9]. Increased expression of IL-10, both at protein and mRNA level, has been detected in OA human cartilage [10], but its role in OA pathogenesis need to be further investigated.

The purpose of this study has been to evaluate the effects of ESW on the metabolism of human OA chondrocytes "in vitro", and particularly on the expression of beta_1 _integrin, IL-10, and TNF-alpha.

## Methods

### Chondrocyte isolation

Human articular cartilage was obtained from nine patients (6 males, 3 females, range age 55–70 years) with primary knee OA, undergoing total joint replacement at our orthopaedic department and from three young healthy donors (HD) (2 males, 1 female, range age 21–37 years) with joint traumatic fracture. We received the approval for our study from the Poloclinical Ethical Commettee of Bari. All patients gave a written informed consent and the study was approved by the local ethical committee. Cartilage was taken from the femoral and tibial sides of knee, minced and chondrocytes were isolated as previously described [10]. Briefly, chondrocytes were released from the cartilage matrix by hyaluronidase (0.2%, 30 min., 37°C, Sigma), pronase (0.25%, 90 min., 37°C, Sigma) and collagenase (0.2%, 3 hours, 37°C, Sigma) sequential enzymatic digestion. More than 95% of the chondrocytes were viable (Trypan blue exclusion test) after their isolation.

### ESW treatment

After isolation, chondrocytes were re-suspended in Dulbecco's modified Eagle medium (DMEM), supplemented with 10% foetal calf serum (FCS) and antibiotics (penicillin 100 UI/ml and streptomycin 100 microg/ml 5% and placed in 1,8 ml cryogenic vials (Nunc, Denmark) at 4–5 × 10^5^/cells/ml. The vials were completely filled with medium and were tested to be permeable for the shock wave. The ESW electromagnetic generator system (MINILITH SL1, STORZ MEDICAL) was used in this study. This ESW device is a focused one, and the location was provided with a ultrasound-guided system. Between the generator and the cells we used an ultrasound gel that was compressed to guaranteed to avoid air. The test-vial was placed in a special cylindrical support on the shockwave generator, which enabled the focal depth probe to be directed correctly to the vial containing the cells. Chondrocytes were subdivided into 5 populations: 4 specimens underwent ESW treatment at different parameters of impulses, energy levels and energy flux density, as shown in Table 1, while an untreated vial was kept as control sample, but underwent the same laboratory processing. The controls vials also were kept outside the incubator for the same time as the treated cells. At the end of the treatment, cellular vitality was evaluated with the Trypan blue dye exclusion test, and chondrocytes were cultured in 24-well plate in DMEM supplemented with 10% FCS for 48 hours prior to flow-cytometry.

**Table 1:** The panel of ESW treatment of chondrocytes.

Chondrocytes	Energy flux density (mJ/mm2)	Energy level	Impulses
A1	0,055	2,5	500
A2	0,055	2,5	1000
B1	0,17	5	500
B2	0,17	5	1000

### Chondrocyte phenotype

After incubation, chondrocytes were re-suspended in PBS containing 0.1% sodium azide and 0.2% bovine serum albumin, and blocked by incubating with 2% normal human serum (Advanced Protein Products, UK). After fixation with paraformaldehyde and permeabilization with saponin (Fix & Perm Cell Permeabilization Kit, Caltag Lab., Burlingame, CA), chondrocytes were incubated (20 min. at 4°C) with 5 microl of FITC/anti-human IL-10 mAb or FITC/anti-human TNF-alpha mAb (Serotec, Oxford, UK). Beta_1 _integrin (Serotec, Oxford, UK) surface expression was assessed with the same procedure without cell membrane permeabilization. Control samples were incubated with rat IgG1-FITC/IgG2-PE (DAKO, Denmark). Stained cells were analyzed on a FACScan (Cell Quest, Becton Dickinson, Mountain View, CA). The FACS setting was identical throughout all the study.

### Statistical analysis

Results are expressed as mean values ± 1 standard deviation (SD). The Student's t test was used to compare the treated chondrocytes with the control group and the level of significance was set at p < 0.05.

## Results

### Cell viability

The Trypan blue dye exclusion test showed that ESW application affected chondrocytes viability. Cell viability was lower in ESW treated chondrocytes than control, both in OA chondrocytes and in normal chondrocytes, although these differences did not reach the significance level. Relevant changes in chondrocyte viability were not seen according to ESW energy levels and pulses applied (Table 2).

**Table 2:** Chondrocyte viability following ESW treatment.

Viability	Energy flux density (mJ/mm2)	Energy level	Impulses
72% ± 10	0,055	2,5	500
69% ± 13	0,055	2,5	1000
77% ± 15	0,17	5	500
74 % ± 18	0,17	5	1000
85 % ± 6	Untreated	-	-

**Figure 1:**
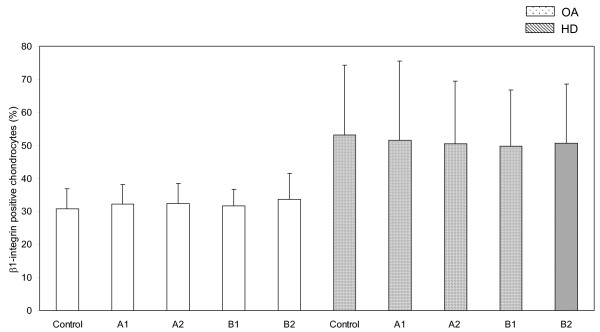
**ESW modulation of expression of beta_1 _integrin on chondrocytes**. Surface expression of beta_1 _integrin on osteoarthritic (OA) and healthy (HD) chondrocytes treated with ESW at different impulses and energies (see table 1 for the panel of ESW treatment) and untreated (control). Bars represents mean ± 1 standard deviation.

As shown on Figure 1, the percentage of chondrocytes bearing beta_1 _integrin was lower in OA (30.8 ± 6) than in normal cartilage (53.2 ± 21) at baseline (p < 0.01), but ESW treatment did not significantly change beta_1 _integrin expression on both OA (A_1 _32.2 ± 6, A_2 _32.4 ± 6, B_1 _31.7 ± 5, B_2 _33.6 ± 8) and normal chondrocytes (A_1 _51.5 ± 24, A_2 _50.4 ± 19, B_1 _49.7 ± 17, B_2 _50.6 ± 18).

**Figure 2:**
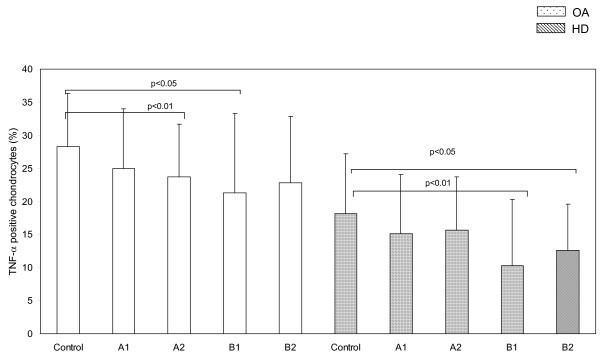
**ESW modulation of intracellular levels of TNF-α in chondrocytes**. Intracellular levels of TNF-α in osteoarthritic (OA) and healthy (HD) chondrocytes treated with ESW at different impulses and energies (see table 1 for the panel of ESW treatment) and untreated (control). Bars represents mean ± 1 standard deviation.

Intracellular levels of TNF-alpha (Figure 2), were significantly higher in OA (28.3 ± 8) than in normal cartilage (18.2 ± 9) at baseline (p < 0.05). ESW application significantly decreased the proportion of chondrocytes expressing intracellular TNF-alpha both in OA (A_1 _25.1 ± 9, A_2 _23.7 ± 8, B_1 _21.3 ± 12, B_2 _22.6 ± 8) and normal cartilage (A_1 _15.1 ± 9, A_2 _11.7 ± 8, B_1 _10.3 ± 10, B_2 _12.6 ± 7).

**Figure 3:**
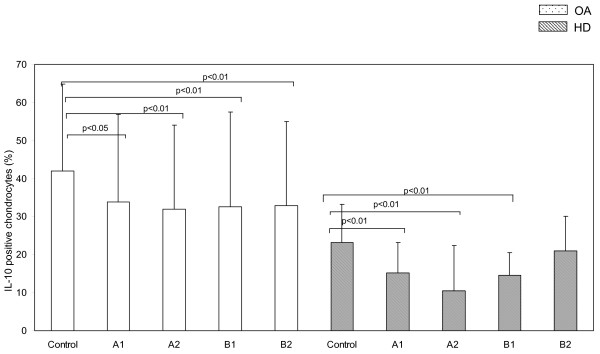
**ESW modulation of intracellular levels of IL-10 in chondrocytes**. Intracellular levels of IL-10 in osteoarthritic (OA) and healthy (HD) chondrocytes treated with ESW at different impulses and energies (see table 1 for the panel of ESW treatment) and untreated (control). Bars represents mean ± 1 standard deviation.

As far as IL-10 concerns, we detected similar results (Figure 3). The percentage of chondrocytes expressing intracellular IL-10 were significantly higher in OA (41.9 ± 23) than in normal cartilage (23.2 ± 10) at baseline (p < 0.01). Following ESW treatment IL-10 levels significantly decreased both in OA (A_1 _33.9 ± 23, A_2 _32 ± 22, B_1 _32.5 ± 25, B_2 _32.9 ± 22) and normal cartilage (A_1 _15.2 ± 8, A_2 _10.5 ± 12, B_1 _14.5 ± 6), except for the strongest ESW application (B_2 _21 ± 9).

## Discussion

In this study we provided evidence that ESW modulate intra-cellular levels of TNF-alpha and IL-10 by human articular chondrocytes both from osteoarthritic patients and healthy controls, without affecting beta1 integrin expression.

Our data confirmed that OA chondrocyte phenotype is defined by reduced beta_1 _integrin expression and increased TNF-alpha and IL-10 levels, as earlier reported [10-12]. The decrease in beta_1 _integrin cell surface expression seems to be an early event in OA pathogenesis and critical in breaking down the complex ECM/chondrocyte interactions that maintain cartilage homeostasis [3]. TNF-alpha plays a crucial role in inducing tissue injury and by interacting with p55 TNF-alpha receptor contributes to focal loss of cartilage in OA [11,13]. TNF-alpha activates MMP and aggrecanase production by chondrocytes [14,15] and cooperates with other cytokines to degrade cartilage matrix [16]. The role of IL-10 on cell metabolism is controversial as having pleiotropyc functions. On one side, IL-10 has proanabolic effects, such as promotion of proteoglycan synthesis by human chondrocytes "in vitro" [17], prevention of cartilage destruction by reducing IL-1 and TNF-alpha mRNA expression in articular chondrocytes [17]; on the other side, IL-10 may also exert procatabolic effects as in human fibroblasts it down-regulates type I collagen mRNA expression and enhances collagenase and stromelysin gene expression [18]. This diversity of responses indicates that downstream events following IL-10 binding may vary depending on the cell target phenotype and on the environment of that cell, thus making the role of IL-10 in OA pathogenesis more intriguing and difficult to understand.

It has been previously shown that ESW induce a dose-dependent increase in cytotoxicity of cultured human chondrocytes assessed by the lactate dehydrogenase (LDH) assay [19], but the possible effects of ESW on the metabolic pathways that are activated in the pathogenetic mechanisms of OA were not evaluated. In our study, ESW application on isolated chondrocytes down-regulated the intracellular levels of TNF-alpha and IL-10, but no changes in β_1 _integrin expression were detected. Furthermore, healthy and OA chondrocytes behaved in a similar fashion. IL-10 expression significantly decreased in chondrocytes treated with all levels of energy and impulses, while TNF-alpha seemed to be selectively reduced at middle levels. These findings might be due to the generic cell damage induced by ESW owing to cell viability reduction following the treatment. However, we believe that those effects are specifically ESW related because cell viability did not correlated to the level of energy exposure, beta_1 _integrin expression did not change after ESW treatment, chondrocytes were cultured 48 hrs after ESW application and before phenotyping their viability rose over 90%.

The relevance of these findings can be only object of speculations at the moment. The reduction of TNF-alpha can be considered as a protective effect and may prevent MMPs activation and cartilage degradation. Understanding of IL-10 changes may be controversial as IL-10 can have both proanabolic and procatabolic effects of tissue metabolism. Additionally, it remains to be clarified whether IL-10 decrease is a specific outcome of ESW treatment or mediated by TNF-alpha reduction since it has been shown that TNF-alpha up-regulates IL-10 expression in rheumatoid arthritis synovitis [20]. At any case, ESW treatment seems to be capable to restore TNF-alpha and IL-10 production by osteoarthritic chondrocytes at normal levels thus potentially interfering with the pathologic mechanisms causing cartilage damage in OA and representing the theoretical rationale for using ESW as therapy of OA.

It has been recently reported that ESW may be useful to treat OA in dogs [1], and veterinarians have begun to use ESW also to treat OA in horses [2]. Our findings seem to provide the biological clue that ESW can be effective in treating OA. Nonetheless, further studies are warranted to implement these preliminary data by evaluating the effects of ESW on other cellular pathways that are critically involved in OA pathogenesis such as the regulation of MMPs, and on other articular tissues affected by OA such as synovial membrane and subchondral bone.

## Conclusion

In our work we have found that ESW application down-regulate the intracellular levels of TNF-alpha and IL-10 by chondrocytes. Considering that in osteoarthritic chondrocytes the expression of beta_1 _integrin is low and TNF-alpha and IL-10 levels are high, we speculate that ESW could restore TNF-alpha and IL-10 production by osteoarthritic chondrocytes at normal levels thus potentially interfering with the pathologic mechanisms causing cartilage damage in OA. However, further in vivo and in vitro studies are necessary to establish if ESW can represent a viable option in the treatment of patients suffering of OA.

## Competing interests

The author(s) declare that they have no competing interests.

## Authors' contributions

BM: proposed the application of ESW on the chondrocytes and took the biopsy tissues during the surgical operations. He also was involved in the revising of the manuscript for the literature content.

FI: gave substantial contributions to conception, design, statistic analysis and interpretation of data.

AN: carried out the treating of the chondrocytes by shock waves and participated to draft the manuscript.

GL: conceived of the study and participated in its coordination and drafting of the manuscript.

LM: carried out the study from the drawing of cartilage tissues to the drafting of the manuscript.

VP: participated in the design and the coordination of the study.

RG: gave the substantial contributes in the drafting the manuscript and in the revising it for the intellectual contents.

All authors read and approved the final manuscript.

## Pre-publication history

The pre-publication history for this paper can be accessed here:


